# An Information-Theoretic Perspective on Proper Quaternion Variational Autoencoders

**DOI:** 10.3390/e23070856

**Published:** 2021-07-03

**Authors:** Eleonora Grassucci, Danilo Comminiello, Aurelio Uncini

**Affiliations:** Department of Information Engineering, Electronics and Telecommunications, Sapienza University of Rome, Via Eudossiana 18, 00184 Rome, Italy; danilo.comminiello@uniroma1.it (D.C.); aurelio.uncini@uniroma1.it (A.U.)

**Keywords:** variational autoencoder, generative learning, quaternion neural networks, properness, second-order circularity

## Abstract

Variational autoencoders are deep generative models that have recently received a great deal of attention due to their ability to model the latent distribution of any kind of input such as images and audio signals, among others. A novel variational autoncoder in the quaternion domain H, namely the QVAE, has been recently proposed, leveraging the augmented second order statics of H-proper signals. In this paper, we analyze the QVAE under an information-theoretic perspective, studying the ability of the H-proper model to approximate improper distributions as well as the built-in H-proper ones and the loss of entropy due to the improperness of the input signal. We conduct experiments on a substantial set of quaternion signals, for each of which the QVAE shows the ability of modelling the input distribution, while learning the improperness and increasing the entropy of the latent space. The proposed analysis will prove that proper QVAEs can be employed with a good approximation even when the quaternion input data are improper.

## 1. Introduction

Generative learning models have been recently gained considerable attention due to their surprising performance in producing highly realistic signals of various types [1,2,3,4]. They have been successfully employed in a wide variety of applications, such as image-to-image translation [5], image fusion [6], face de-identification [7], natural language generation [8], data augmentation on ancient handwritten characters [9], MRI super-resolution [10], brain tumor growth prediction [11], generative modeling of structured-data [12].

Among such generative methods, the variational autoencoders (VAEs) have been proven to perform stochastic variational inference and learning even for large datasets and intractable posterior distributions [13]. The main advantages of the VAEs rely on their capability of learning smooth latent representations of the input data. This has led VAEs to be used in several fields of applications, including high-quality image generation [14], speech enhancement [15], music style transfer [16], data augmentation [17], 3D scene generation [18], gesture similarity analysis [19], text generation [20] and sequential recommendation [21], among others.

Recent advances on VAEs focus both on theoretical aspects, such as the improvement of the stochastic inference approach [22], and on architectural aspects, such as the use of different types of latent variables to learn local and global structures [23], the definition of hierarchical schemes [24], rather than the use of a multi-agent generator [25]. Among the most recent VAE models, we focus on the quaternion-valued variational autoencoder (QVAE), which exploits the properties of quaternion algebra to improve performance, on one hand, and to significantly reduce the overall number of network parameters [26], on the other hand.

The basic approach on which relies the QVAE is the learning in the quaternion domain, which results in significant advantages in the presence of multidimensional input data (mainly 3D and 4D data) showing some inter-channel correlations. This properties have been widely exploited in shallow learning models, such as linear and nonlinear adaptive filters [27,28,29,30,31,32,33,34]. Another fundamental property of quaternion-valued learning is the Hamilton product, which has recently favored the proliferation of convolutional neural networks in the quaternion domain [35,36,37,38]. Due to their capabilities, quaternion-valued learning methods have been applied in several applications, including spoken language understanding [39], color image processing [40,41], 3D audio [42,43], speech recognition [44], image generation [45], quantum mechanics [46], risk diversification [47], gait data analysis [48].

As with any other complex-valued and hypercomplex-valued algebra, the quaternion-valued learning can rely on the properties of the second-order statistics, which are fundamental to characterize the input data based on their correlation. A characterization of correlation is achieved in terms of covariance and pseudo-covariance. In particular, random variables and processes with a vanishing pseudo-covariance are called proper [49,50,51]. Properness is preserved under affine transformations. Moreover, it should be considered that the multivariate Gaussian density assumes a natural form only for proper random variables. Properness in the quaternion domain, also denoted as H-properness, is based on the vanishing of three different complementary covariance matrices [51]. In the case of possibly improper random variables, we need to take into account the quaternion conjugate and augmented second-order statistics [51,52,53]. The degree of improperness of a quaternion random vector can be measured in several ways. An interesting approach is to use an improperness measure based on the Kullback–Leibler (KL) divergence between the augmented covariance matrix and its closest proper version in the KL sense [51]. Quaternion second-order statistics have been used in several applications, from independent component analysis to canonical transform [54,55], from linear to nonlinear adaptive filtering [56,57,58].

Recently, augmented quaternion second-order statistics have been also exploited for the first time for the development of a deep learning model, the QVAE [26]. Deep VAEs should model long-range correlations in data [59] and quaternion layers allow to leverage internal latent relations between input features. The QVAE also leads to a significant reduction in the number of parameters, which may benefit the decoder. A full improper QVAE should be developed to completely exploit correlated multidimensional input data, such as color images. However, on one hand an improper QVAE may be too complex due to the sophisticated structure of the complete covariance matrix. On the other hand, the proper QVAEs may represent a good tradeoff between complexity and performance, even when input data are improper. To this end, an information-theoretic analyses, thanks to its relation with improperness measures, may help in understanding how good such approximation may be in terms of properness [60,61].

In order to understand and demystify the theoretical capabilities of generating data with a proper model when the input data is either proper or improper, in this paper we investigate the proper QVAE from an information-theoretic perspective. To this end, we show how the KL divergence may establish a relation between the loss function of the QVAE and the improperness measure. We exploit the KL-based measure to show the entropy loss due to the improperness of the quaternion random vector input of the QVAE, or equivalently, the mutual information among the quaternion random vector and its involutions over three pure unit quaternions [51,52]. We show how the entropy in input data plays an important role in the generation of improper signals. Furthermore, we illustrate how the improperness can be calibrated in the latent space in order to enhance its differential entropy. Simulation results on artificial signals will prove the ability of the proper QVAE to be adopted with a good approximation for the generation of improper signals. Moreover, results will show how the properness is preserved by the model, even if it is composed of several nonlinearities.

The paper is organized as follows. In Section 2, we briefly introduce the main properties of quaternion algebra that are exploited in quaternion-valued processing. In Section 3, we analyze the quaternion properness and the augmented second-order statistics of quaternion-valued signals, which are then exploited to define the quaternion variational autoencoders. In particular, the proper QVAE is described in Section 4, while in Section 5 we define the relation between the loss function of the QVAE and the properness measures of the involved signals by using the quaternion-valued entropy. Experimental simulations are shown in Section 6, while final conclusions are derived in Section 7.

## 2. Fundamental Properties of Quaternion Algebra

Quaternions are hypercomplex numbers consisting of four components, a scalar part and three imaginary ones, and elements of $R^{4}$: The quaternion domain H is an extension of the complex domain C. A quaternion is identified as

(1) $$q=q_{0}+q_{1}\hat{ı}+q_{2}ĵ+q_{3}\hat{\kappa }=q_{0}+q$$

in which $q_{0},\;q_{1},\;q_{2},\;q_{3}$ are real coefficients and $\hat{ı},\;ĵ,\;\hat{\kappa }$ imaginary units. A quaternion without the scalar part $q_{0}$ is named *pure quaternion* and represents a vector in $R^{3}$ since the imaginary units play the role of orthonormal basis, being $\hat{ı}=\left(1,0,0\right),\;ĵ=\left(0,1,0\right),\;\hat{\kappa }=\left(0,0,1\right)$. The quaternions’ peculiarity is the relation among imaginary components which comply with the following properties:

(2) $$\begin{array}{c} {\hat{ı}}^{2}={ĵ}^{2}={\hat{\kappa }}^{2}=-1\\ \hat{ı}ĵ=\hat{ı}\times ĵ=\hat{\kappa };\;ĵ\hat{\kappa }=ĵ\times \hat{\kappa }=\hat{ı};\;\hat{\kappa }\hat{ı}=\hat{\kappa }\times \hat{ı}=ĵ\\ \hat{ı}ĵ=-ĵ\hat{ı};\;ĵ\hat{\kappa }=-\hat{\kappa }ĵ;\;\hat{\kappa }\hat{ı}=-\hat{ı}\hat{\kappa }. \end{array}$$

The fundamental properties of quaternion operations are then described below.

**Addition and subtraction of two quaternions.** Addition and subtraction among quaternions *q* and *p* are performed component-wise:

(3) $$q\pm p=q_{0}\pm p_{0}+\left(q_{1}\pm p_{1}\right)\hat{ı}+\left(q_{2}\pm p_{2}\right)ĵ+\left(q_{3}\pm p_{3}\right)\hat{\kappa }.$$

**Scalar product of two quaternions.** As for addition and subtraction, the scalar product is also an element-wise operation:

(4) $$q\cdot p=q_{0}p_{0}+q_{1}p_{1}+q_{2}p_{2}+q_{3}p_{3}.$$

**Vector product of two quaternions.** Due to the relations among imaginary units in (2) and the non commutative properties of vector product in the hypercomplex domain, a new kind of vector multiplication has to be introduced. The Hamilton product, denoted by ⊗, is at the core of quaternion neural networks and it is described by the following equation:

(5) $$\begin{array}{cc} q⊗p & =\left(q_{0}+q_{1}\hat{ı}+q_{2}ĵ+q_{3}\hat{\kappa }\right)\left(p_{0}+p_{1}\hat{ı}+p_{2}ĵ+p_{3}\hat{\kappa }\right)\\ \; & =\left(q_{0}p_{0}-q_{1}p_{1}-q_{2}p_{2}-q_{3}p_{3}\right)\\ \; & +\left(q_{0}p_{1}+q_{1}p_{0}+q_{2}p_{3}-q_{3}p_{3}\right)\hat{ı}\\ \; & +\left(q_{0}p_{2}-q_{1}p_{3}+q_{2}p_{0}+q_{3}p_{2}\right)ĵ\\ \; & +\left(q_{0}p_{3}+q_{1}p_{2}-q_{2}p_{1}+q_{3}p_{0}\right)\hat{\kappa }. \end{array}$$

The Hamilton product can also be written in concise form:

$$q⊗p=q_{0}p_{0}-q\cdot p+q_{0}p+p_{0}q+q\times p,$$

from which it is straightforward obtaining the Hamilton product for pure quaternions, by fixing $q_{0}=p_{0}=0$:

q⊗p=−q·p+q×p.

**Conjugate of a quaternion.** The conjugate of a quaternion number is obtained by subtracting the imaginary units instead of adding them to the scalar part:

(6) $$q^{*}=q_{0}-q_{1}\hat{ı}-q_{2}ĵ-q_{3}\hat{\kappa }=q_{0}-q.$$

**Norm of a quaternion.** The norm of a quaternion is simply the euclidean norm in $R^{4}$, thus $\left|q\right|=\sqrt{qq^{*}}=\sqrt{q_{0}^{2}+q_{1}^{2}+q_{2}^{2}+q_{3}^{2}}$.

**Quaternion polar form.** Quaternions admit an Euler representation, so considering a quaternion *q*, an angle θ∈R and a pure unit quaternion $\nu =q/∥q∥$, the polar form is:

$$q=|q|\left(\cos\left(\theta \right)+\nu \sin\left(\theta \right)\right)=\left|q\right|e^{\nu \theta }$$

in this setting, $\cos\left(\theta \right)=q_{0}/∥q∥$ and $\sin\left(\theta \right)=∥q∥/∥q∥$.

**Quaternion involutions.** A quaternion involution is generally defined as a mapping which is its own inverse or self-inverse mapping. Quaternions have infinite involutions that can be generalized by [62]:

(7) $$q^{\nu }=-\nu q\nu$$

in which *q* is the quaternion to be involved and ν is the axis of the involution. Interestingly, the scalar part of the quaternion is invariant to any kind of involution while the imaginary parts are reflected across the axis of the involution. Consequently, the conjugate of a quaternion is an involution since the scalar part remains unchanged while the imaginary units are reflected through the axis. Together with the conjugate, there are three crucial involutions we have to consider, which are perpendicular involutions:

(8) $$\begin{array}{cc} \; & q^{\hat{ı}}=-\hat{ı}q\hat{ı}=q_{0}+q_{1}\hat{ı}-q_{2}ĵ-q_{3}\hat{\kappa }\\ \; & q^{ĵ}=-ĵqĵ=q_{0}-q_{1}\hat{ı}+q_{2}ĵ-q_{3}\hat{\kappa }\\ \; & q^{\hat{\kappa }}=-\hat{\kappa }q\hat{\kappa }=q_{0}-q_{1}\hat{ı}-q_{2}ĵ+q_{3}\hat{\kappa } \end{array}$$

In the perpendicular involutions, the quaternion is involved across its imaginary units.

## 3. Augmented Second-Order Statistics and Quaternion Properness

### 3.1. Statistics for Quaternion Random Signals

In order to understand the behavior of a signal, it is often crucial to study its statistics. A generic Gaussian signal can be completely described by the mean and the covariance matrix. For quaternion random variables, the mean can be easily derived by (3), resulting in another quaternion:

(9) $$\mu_{q}={\bar{q}}_{0}+{\bar{q}}_{1}\hat{ı}+{\bar{q}}_{2}ĵ+{\bar{q}}_{3}\hat{\kappa }$$

in which ${\bar{q}}_{\delta },\;\delta =\left\{0,1,2,3\right\}$ is the average of each quaternion component. Conversely, deriving second-order statistics for quaternion signals requires more detailed computations due to the interactions among components. As previously demonstrated for complex numbers [49], the covariance matrix is not able to describe the second-order information and the complementary covariance matrix needs to be considered too. Consequently, taking the standard covariance matrix of a quaternion signal into account is not sufficient to cover the complete second-order statistics; thus, the information is augmented through three complementary covariance matrices [52]. In order to build this complete statistical description, we need to introduce the information brought by the perpendicular involutions $q^{\hat{ı}},\;q^{ĵ},\;q^{\hat{\kappa }}$ introduced in (8). The matrices to be considered are then:

(10) $$\begin{array}{c} C_{qq}=E\left\{qq^{H}\right\},\;C_{qq^{\hat{ı}}}=E\left\{qq^{{\hat{ı}}^{H}}\right\},\;C_{qq^{ĵ}}=E\left\{qq^{{ĵ}^{H}}\right\},\;C_{qq^{\hat{\kappa }}}=E\left\{qq^{{\hat{\kappa }}^{H}}\right\} \end{array}$$

which are, respectively, the standard covariance matrix and the three complementary matrices. Note that ${\left(\cdot \right)}^{H}$ is the conjugate transpose operator. These matrices are still quaternions, so they comprise a real part and three imaginary units. As an example, we analyze the first matrix $C_{qq}$. It is composed by the covariance matrices of the quaternion components $q_{0},\;q_{1},\;q_{2},\;q_{3}$ as follows:

(11) $$\begin{array}{cc} C_{qq} & =\left(C_{q_{0}}+C_{q_{1}}+C_{q_{2}}+C_{q_{3}}\right)\\ \; & +\left(C_{q_{1}q_{0}}-C_{q_{0}q_{1}}+C_{q_{3}q_{2}}-C_{q_{2}q_{3}}\right)\hat{ı}\\ \; & +\left(C_{q_{2}q_{0}}-C_{q_{0}q_{2}}+C_{q_{1}q_{3}}-C_{q_{3}q_{1}}\right)ĵ\\ \; & +\left(C_{q_{3}q_{0}}-C_{q_{0}q_{3}}+C_{q_{2}q_{1}}-C_{q_{1}q_{2}}\right)\hat{\kappa }. \end{array}$$

Similarly, also the matrices $C_{qq^{\hat{ı}}},\;C_{qq^{ĵ}},\;C_{qq^{\hat{\kappa }}}$ can be determined and written in the above quaternion form.

The composition and the combination of the standard covariance matrix and of the complementary covariance matrices give rise to the augmented covariance matrix ${\tilde{C}}_{qq}$ which recovers the complete second-order information of the augmented quaternion vector $\tilde{q}=\left[q,q^{\hat{ı}},q^{ĵ},q^{\hat{\kappa }}\right]$[52]. The matrices in (10) have interesting properties: each component of the matrix is symmetric but the component corresponding to the involved imaginary unit is, instead, skew-symmetric. These characteristics are included in the Hermitian property, according to which $C_{qq^{\hat{ı}}}$ is $\hat{ı}$-Hermitian, $C_{qq^{ĵ}}$ is ĵ-Hermitian and $C_{qq^{\hat{\kappa }}}$ is $\hat{\kappa }$-Hermitian. The derived augmented covariance matrix follows.

(12) $${\tilde{C}}_{qq}=E\left\{\tilde{q}{\tilde{q}}^{H}\right\}=\left[\begin{array}{cccc} C_{qq} & C_{qq^{\hat{ı}}} & C_{qq^{ĵ}} & C_{qq^{\hat{\kappa }}}\\ C_{qq^{\hat{ı}}}^{H} & C_{q^{\hat{ı}}q^{\hat{ı}}} & C_{q^{\hat{ı}}q^{ĵ}} & C_{q^{\hat{ı}}q^{\hat{\kappa }}}\\ C_{qq^{ĵ}}^{H} & C_{q^{ĵ}q^{\hat{ı}}} & C_{q^{ĵ}q^{ĵ}} & C_{q^{ĵ}q^{\hat{\kappa }}}\\ C_{qq^{\hat{\kappa }}}^{H} & C_{q^{\hat{\kappa }}q^{\hat{ı}}} & C_{q^{\hat{\kappa }}q^{ĵ}} & C_{q^{\hat{\kappa }}q^{\hat{\kappa }}} \end{array}\right].$$

By means of the mean and augmented covariance matrix and following [52], it is now possible to define a generic quaternion Gaussian distribution $p\left(\tilde{q}\right)=p\left(q,q^{\hat{ı}},q^{ĵ},q^{\hat{\kappa }}\right)$, which is Gaussian if its components are jointly normal:

(13) $$p\left(\tilde{q}\right)=\frac{1}{{\left(\pi /2\right)}^{2N}\det{\left({\tilde{C}}_{qq}\right)}^{1/2}}\exp\left\{-\frac{1}{2}{\left(\tilde{q}-{\tilde{\mu }}_{q}\right)}^{H}{\tilde{C}}_{qq}^{-1}\left(\tilde{q}-{\tilde{\mu }}_{q}\right)\right\},$$

where the mean ${\tilde{\mu }}_{q}$ is the mean of the augmented quaternion $\tilde{q}$ and *N* the number of samples.

### 3.2. H-Properness

In the previous subsection, we describe the complete second-order information of a quaternion signal by means of the augmented covariance matrix which is comprised of the covariance matrices of the quaternion with its perpendicular involutions. Due to the four degrees of freedom in quaternions, the H domain includes manifold properness definitions that are employed to characterize the relation of q with $q^{\hat{ı}},\;q^{ĵ},\;q^{\hat{\kappa }}$ in some particular cases.

The lighter condition is described in the R-properness, which holds for a signal that is uncorrelated with one of its perpendicular involutions, leading to the cancellation of either one of the complementary covariance submatrices $C_{qq^{\hat{ı}}},\;C_{qq^{ĵ}},\;C_{qq^{\hat{\kappa }}}$. A slightly harder constraint is brought by the C-properness which claims the nullification of two of the three complementary covariance submatrices. A composition of the two already defined properties gives rise to the strongest properness condition, the H-properness. The latter holds when each complementary covariance matrix vanishes, that is $C_{qq^{\hat{ı}}}=C_{qq^{ĵ}}=C_{qq^{\hat{\kappa }}}=0$. However, according to [52], an H-proper variable has also several other features that are reported in Table 1. An H-proper signal is then uncorrelated with its perpendicular involutions and, according to the second property in Table 1, it also has uncorrelated components $q_{0},\;q_{1},\;q_{2}\;q_{3}$ which have equal variance $\sigma^{2}$. From these assumptions, we can derive the corresponding augmented covariance matrix for H-proper signals which will be symmetric and positive definite:

(14) $${\hat{C}}_{qq}=E\left\{\tilde{q}{\tilde{q}}^{H}\right\}=\left[\begin{array}{cccc} C_{qq} & 0 & 0 & 0\\ 0 & C_{q^{\hat{ı}}q^{\hat{ı}}} & 0 & 0\\ 0 & 0 & C_{q^{ĵ}q^{ĵ}} & 0\\ 0 & 0 & 0 & C_{q^{\hat{\kappa }}q^{\hat{\kappa }}} \end{array}\right]=4\sigma^{2}I.$$

The structure of the matrix ${\hat{C}}_{qq}$ is invariant to any linear transformation $\nu =T^{{}^{H}}q$ where T is a quaternion weight matrix [54], thus ensuring that the quaternion properness is preserved by linear layers in neural networks.

As for improper signals in Section 3.1, a probability density function can be derived for H-proper signals too. In this framework, the distribution is described by the covariance matrix in (14) which is equal to $4\sigma^{2}I$ and by the mean of the quaternion component in (9):

(15) $$p\left(\tilde{q}\right)=\frac{1}{{\left(2\pi \sigma^{2}\right)}^{2N}}\exp\left\{-\frac{1}{2\sigma^{2}}{\left(q-\mu_{q}\right)}^{H}\left(q-\mu_{q}\right)\right\}$$

In order to evaluate the properness of an augmented zero-mean quaternion random vector, a simple and fast coefficient was proposed in [32]. It indicates the correlation of the signal with its perpendicular involutions through the complementary covariance matrices. Note that the coefficient is bounded in [0,1] and it is 0 in the case of H-proper signals, while it is equal to 1 for improper ones. It is defined as

(16) $$I_{\alpha }=\left|\frac{C_{qq^{\alpha }}}{C_{qq}}\right|,\;\;\;\alpha =\hat{ı},ĵ,\hat{\kappa }.$$

In our experiments, since we are dealing with H-proper signals, we can just report the average of the three coefficients, simply denoted as *I*. Conversely, if we evaluate R or C proper signals, we should consider each coefficient independently.

## 4. The H-Proper Variational Autoencoder

Originally introduced in [26] (the implementation of the QVAE is available online at: https://github.com/eleGAN23/QVAE, accessed on 1 July 2021), the quaternion variational autoencoder (QVAE) is a generative model which learns and controls the latent distribution of an *n*-dimensional H-proper quaternion input. Once the distribution is learnt, then the model is able to generate new samples from it thanks to the probabilistic relation between the two quaternion spaces of the input and of the latent distribution.

Similar to the variational autoencoder (VAE) for real-valued distributions in [13], the QVAE grasps the mapping of the original quaternion input x∈H into the latent space described by a prior distribution $p_{\theta }\left(\epsilon \right)$, with ϵ∈H. Then, it learns the conditional distribution $p_{\theta }\left(x|z\right)$ of the input with respect to the latent vector z∈H, that is the prior distribution adjusted with the statistics learnt by the encoder. However, due to the intractability of the mapping distribution $p_{\theta }\left(z|x\right)$, the QVAE introduces an approximation $q_{\phi }\left(z|x\right)$ to encode the input into the latent vector, as in its real-valued counterpart [13]. The parameters of this recognition model (or inference model) are then optimized to match the true intractable conditional distribution as $q_{\phi }\left(z|x\right)\approx p_{\theta }\left(z|x\right)$. By employing this approximation, it is possible to express the marginal likelihood

(17) $$\begin{array}{cc} \log\left(p_{\theta }\left(x\right)\right) & =\lambda D_{\mathrm{KL}}\left(q_{\phi }\left(z|x\right)∥p_{\theta }\left(z|x\right)\right)+L_{\theta ,\phi }\left(x\right)\\ \; & =-\lambda D_{\mathrm{KL}}\left(q_{\phi }\left(z|x\right)∥p_{\theta }\left(\epsilon \right)\right)+E_{q}\left\{\log\left(p_{\theta }\left(x|z\right)\right)\right\} \end{array}$$

where $L_{\theta ,\phi }\left(x\right)$ is the so called *evidence lower bound* (ELBO) [13] with respect to the parameters ϕ and θ, which is a lower bound on the log-likelihood of the data distribution [63]. $D_{\mathrm{KL}}\left(\cdot \right)$ is the KL divergence between the H-proper centered isotropic Gaussian variable $p_{\theta }\left(\epsilon \right)∼N\left(0,{\hat{C}}_{\epsilon \epsilon }\right)$ and the H-proper Gaussian $q_{\phi }\left(z|x\right)∼N\left(\mu_{z},{\hat{C}}_{zz}\right)$, with ${\hat{C}}_{zz}$ taking the form of (14) and ${\hat{C}}_{\epsilon \epsilon }=4I\sigma^{2}$. The KL divergence in the quaternion domain takes the following form:

(18) $$\begin{array}{cc} D_{\mathrm{KL}}\left(q_{\phi }\left(z|x\right)∥p_{\theta }\left(\epsilon \right)\right)= & \frac{1}{2}\left(\mathrm{Tr}\left\{{\hat{C}}_{\epsilon \epsilon }^{-\frac{1}{2}}{\hat{C}}_{zz}{\hat{C}}_{\epsilon \epsilon }^{-\frac{1}{2}}\right\}\right.\\ \; & +\left.\mu_{z}^{H}{\hat{C}}_{\epsilon \epsilon }^{-1}\mu_{z}-N+\log\left(\frac{\det\left({\hat{C}}_{\epsilon \epsilon }\right)}{\det\left({\hat{C}}_{zz}\right)}\right)\right). \end{array}$$

The optimization of the QVAE cost function (17) goes through the minimization of the KL divergence, through which the QVAE forces the recognition model to be as close as possible to the prior distribution by operating on the statistics $\mu_{z}$ and ${\hat{C}}_{zz}$.

## 5. Kullback–Leibler Divergence as Entropy Loss Due to Improperness

The KL divergence in (17) can be indeed seen as a measure of the improperness of the recognition model. As defined in [51], the measure is described by:

(19) $$P=\min_{{\hat{C}}_{\epsilon \epsilon }\in C}D_{\mathrm{KL}}\left({\hat{C}}_{zz}∥{\hat{C}}_{\epsilon \epsilon }\right)$$

where C is the set of all the augmented covariance matrices respecting one of the quaternion propernesses introduced in Section 3.2. This measure estimates the distance of the recognition model $q_{\phi }\left(z|x\right)$ with an augmented covariance matrix ${\hat{C}}_{zz}$ from the H-proper isotropic Gaussian variable $p_{\theta }\left(\epsilon \right)$ described by the augmented covariance matrix ${\hat{C}}_{\epsilon \epsilon }$, by assuming $\mu_{z}=0$.

Since we are considering distributions with zero mean, the first term in the second line of the KL Equation (17) vanishes. Furthermore, Equation (18) minimizes the divergence over the set C; thus, in the H-proper case, the matrix which minimizes (18) is the diagonal covariance matrix defined in (14). Employing these considerations and with some computations, we can reduce the improperness measure to:

(20) $$P=-\frac{1}{2}\log\left(\det\left\{{\hat{C}}_{\epsilon \epsilon }^{-\frac{1}{2}}{\hat{C}}_{zz}{\hat{C}}_{\epsilon \epsilon }^{-\frac{1}{2}}\right\}\right).$$

If the signal for which we want to measure the improperness, i.e., the recognition model $q_{\phi }\left(z|x\right)$, is H-proper, then the measure will be close to 0 since ${\hat{C}}_{zz}$ will be close to ${\hat{C}}_{\epsilon \epsilon }$ in the KL sense. Conversely, if the signal is improper, P will be far from 0.

By defining the improperness measure through the KL, it is possible to leverage the relation among the divergence and the entropy and exploring the mutual information of the signal. Therefore, we can use the definition of the differential entropy for a generic quaternion vector [51,52] to compute the entropy of the recognition model $q_{\phi }\left(z|x\right)$:

(21) $$\begin{array}{cc} H\left(q_{\phi }\left(z|x\right)\right) & =2N\log\left(\frac{\pi e}{2}\right)+\frac{1}{2}\log\left(\det\left({\hat{C}}_{zz}\right)\right)\\ \; & =\log\left({\left(\frac{\pi e}{2}\right)}^{2N}\det{\left({\hat{C}}_{zz}\right)}^{\frac{1}{2}}\right). \end{array}$$

The entropy expression can be simplified for isotropic H-proper variables such as the prior distribution $p_{\theta }\left(\epsilon \right)$, by replacing the value of the determinant from (14), which is equal to ${\left(4\sigma^{2}\right)}^{4N}$.

(22) $$\begin{array}{cc} H\left(p_{\theta }\left(\epsilon \right)\right) & =\log\left({\left(\frac{\pi e}{2}\right)}^{2N}{\left({\left(4\sigma^{2}\right)}^{4N}\right)}^{\frac{1}{2}}\right)\\ \; & =2N\left(\log\left(2\pi e\sigma^{2}\right)\right). \end{array}$$

In [52], the authors proved that the differential entropy is maximum for H-proper signals. Thus, (21) is an upper bound for the general entropy definition (20), hence: $H\left(q_{\phi }\left(z|x\right)\right)\leq H\left(p_{\theta }\left(\epsilon \right)\right)$.

Then, according to [51], the improperness measure P can be rewritten through the difference between the entropy of the generic augmented quaternion and the entropy of the isotropic variable:

(23) $$P_{H}=H\left(p_{\theta }\left(\epsilon \right)\right)-H\left(q_{\phi }\left(z|x\right)\right).$$

This formulation elegantly evaluates the loss of entropy that is due to the improperness of the recognition model. Therefore, we can measure the properness of the recognition model and compute the QVAE entropy loss due to the measure of properness (or improperness) of $q_{\phi }\left(z|x\right)$. Thus, when optimizing the QVAE cost function during training, together with the ELBO minimization, through the KL divergence we are jointly minimize also the difference between the Gaussian distributions $p_{\phi }\left(\epsilon \right)$ and $q_{\phi }\left(z|x\right)$ and the entropy loss of the latter.

Since the KL under the information theoretic perspective allows the QVAE to deal also with improper signals, although the QVAE is originally set up for H-proper distributions, by means of an approximation it can be employed also for improper random variables. Let us consider an incoming improper signal x∈H, so both the recognition model $q_{\phi }\left(z|x\right)$ and the generative model $p_{\theta }\left(x|z\right)$ are represented by improper distributions too. We leave instead unchanged the prior distribution $p_{\theta }\left(\epsilon \right)$, which is the centered isotropic H-proper distribution. On the training stage, the KL divergence of the QVAE will move the improper distribution towards the H-proper prior distribution. While the distribution learning is well performed by the model, the KL takes care of increasing the entropy by slightly reducing the improperness. However, the overall original (improper) structure of the signal is preserved by the QVAE which just aims at enhancing the entropy of the recognition model, while leaving free the generative model.

## 6. Experimental Results

In this section, we report the sets of experiments we performed for testing the reliability of the improperness measures and the performances of the H-proper variational autoencoder in learning proper as well as improper distributions.

### 6.1. Proper and Improper Test Signals

We consider two proper signals and two improper signals and we evaluate the improperness coefficient (16), the differential entropy (20), the improperness measure (18) and the entropy loss (22). We expect that the proper signals have improperness measure and coefficients close to 0, as well as the entropy loss. On the contrary, the entropy should be greater than for the corresponding improper signals.

As input signals, we consider:

1. H-proper signal x with independent Gaussian components defined as:
  $$P1:\;x_{\delta }∼N\left(0.03,1\right)$$
  with δ={0,1,2,3}.
2. H-proper filtered signal x from a colored Gaussian noise η[n] as [31]:
  $$P2:\;x\left[n\right]=bx\left[n-1\right]+\frac{\sqrt{1-b^{2}}}{\sqrt{4}}\eta \left[n\right]$$
  with b=0.5.
3. Improper signal x from a quaternion q with components:
  $$q_{0}=0,\;q_{1}∼N\left(0,1\right),\;q_{2}∼N\left(0,1\right),\;q_{3}∼N\left(0,1\right)\;$$
  and composed as:
  $$I1:\;x=ze^{q},\;\;\mathrm{with}\;z∼N\left(0.03,1\right)$$
4. Improper filtered signal x from a Gaussian noise η[n] as:
  $$I2:\;x_{\delta }\left[n\right]=\sqrt{1-b_{\delta }^{2}}+\left(1-b_{\delta }\right)\eta \left[n\right]$$
  with b=[0.75,0.78,0.82,0.85] and δ={0,1,2,3}.

Mild improper signals (i.e., with $I_{\alpha }$ different from 0 and to 1) can be generated by varying the coefficient *b* in the improper signal I2. As an example, by setting b=[0.01,0.2,0.75,0.99], it is possible to obtain a mild-improper signal with $0.4<I_{\alpha }<0.7$. However, we want to stress the H-proper QVAE with completely improper cases that are the extreme and most difficult cases. For this reason, we only consider improper signals with $I_{\alpha }=1$.

We compute the measures defined in Section 5 for each of these signal by using the Quaternion Toolbox for MATLAB${}^{®}$ (S. J. Sangwine and N. Le Bihan. Quaternion Toolbox for MATLAB${}^{®}$, Version 2 with support for Octonions, 2013. First public release 2005. Software library available online at: http://qtfm.sourceforge.net/, accessed on 1 July 2021) and report the results in Table 2. As we expect, the proper signals have low improperness coefficients, meaning that the complementary covariances are sparse, while the coefficients for the improper signals are reasonably equal to 1. On the other hand, the differential entropy is low and negative for the improper signals, while it is highest for the proper ones, proving that (21) is an upper bound for the generic differential entropy. Moreover, the entropy loss is almost 0 for proper signals, confirming that there is no loss of entropy due to the improperness. On the contrary, the improper signals come at the cost of a high entropy drop.

### 6.2. Proper and Improper Signals for an H-Proper QVAE

**Figure 1:**
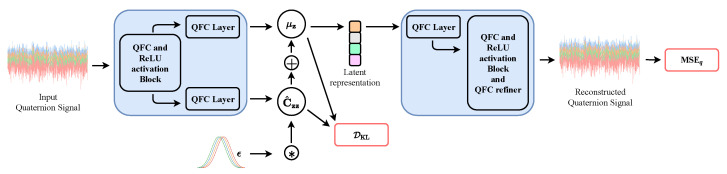
Plain QVAE architecture. The input signal can be either proper or improper. The encoder network is composed of quaternion fully connected layers (QFC) and it learns the statistics of the original distribution and employs them to build the latent representation, on which the KL divergence acts. The representation is then passed to the decoder network which reconstruct the input signal, evaluating the result by means of a quaternion MSE loss (MSE${}_{q}$).

We consider a plain QVAE with a quaternion encoder network and a quaternion decoder network. The first model is comprised of a stack of five quaternion fully connected layers (QFC) with an increasing number of weights [32,64,128,256,512], as in [26,64] and split ReLU activation function as the PixelHVAE in [65]. The statistics of the latent distribution are learnt through two additionally quaternion linear layers with number of weights equal to 4 times the latent dimension, which we fix to 25. The decoder has a mirrored structure, and thus quaternion fully connected layers are piled up as [512,256,128,64,32], with an additional refiner layer at the end of the stack. We do not consider including the quaternion batch normalization [38,45] since it can introduce randomness which may affect the correct learning of the distribution statistics [24,66]. For every experiment, the prior distribution is a centered isotropic H-proper quaternion Gaussian distribution, as described in Section 4. We employ a training signal with 1000 samples, a validation set of 500 and a test set of 1000 without mini batches and we perform 5k iterations with Adam having a learning rate equal to 0.0005. As in the original QVAE [26], the cost function is a weighted sum of a quaternion mean squared error as defined in [37], and the KL divergence of Section 5, for which we fix the scale parameter λ equal to 0.1. Figure 1 shows the architecture of the QVAE we consider in our tests.

**Figure 2:**
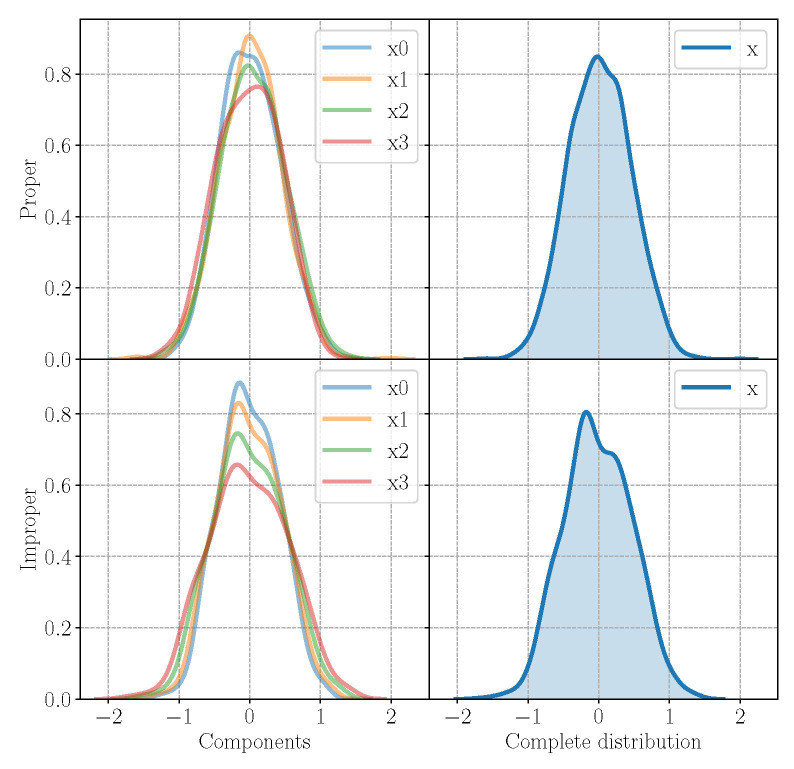
Generated samples from the QVAE trained on the proper signal P1 (first line), and on the improper signal I2 (second line). On the left are the generated components, on the right are the complete distribution of the signal. While H-proper components have the same variance, the improper ones have different variance values, respecting the properties of the original signal.

**Figure 3:**
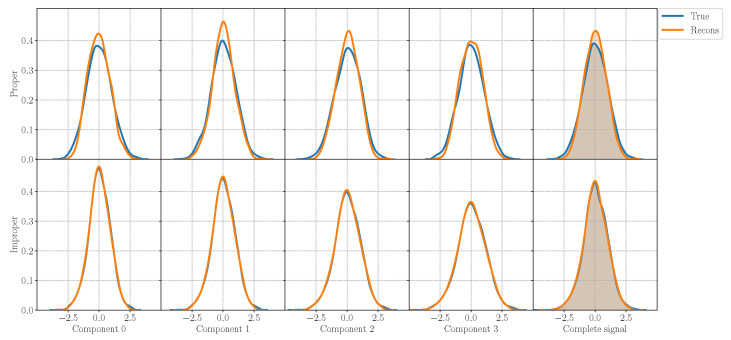
Original and reconstructed distributions from the QVAE trained with the proper signal P1 in the first line, and with improper signal I2 in the second line. The QVAE is able to perfectly learn the different variance values for each of the four components, thus fully reconstructing the distribution of the input signal.

For each experiment, we evaluate the performance of the model in the reconstruction task and generation task as well as on the ability of preserving the properness or the improperness of the incoming signal. In order to assess the generation ability of the model, in Figure 2, we plot the generated components and the complete signal sampling from the model trained on a proper signal and on an improper signal. While the components $x_{0},x_{1},x_{2},x_{3}$ of the improper signal show different variances, the ones from the proper signal has the same variance according to the properties in Table 1. Figure 3 reports instead the original and reconstructed distributions for the proper and improper case, in the first and in the second line, respectively, with an MSE equal to 0.1161 for the proper signal and to 0.01293 for the improper one. In the figure, the first four columns represent the four components $x_{0},x_{1},x_{2},x_{3}$, while the last one displays the distribution of the complete signal x. The H-QVAE is able to perfectly reconstruct the input distribution even in the improper case, grasping the different variances in each component and learning the complete signal. Thus, the QVAE is effective even if approximated for improper distributions, for which it is capable of learning the correct statistics of each component.

Table 3 presents the results of the improperness and entropy analysis on the reconstructed and generated signals on the test set. As for the original samples in Table 2, we compute the improperness coefficient (16), the differential entropy (20), the improperness measure (18) and the entropy loss (19). It is worth noting that the QVAE trained on H-proper signals still generates and reconstruct H-proper signals, as measured by the improperness measures and the entropy loss. Moreover, the entropy of this kind of signal is higher than the entropy of the improper samples. While we already knew that H-properness is preserved by linear transformations, this result asserts that it is maintained also for non-linear transformations brought by activation functions in neural networks, adding an important milestone in the study of H-proper signals.

Another interesting result comes when the QVAE is trained on improper signals. While the improperness coefficients of both reconstructed and generated signals are close to the coefficients of their respective original signals in Table 2, indicating the improperness of these random variable, the entropy values and losses values are intriguing. Indeed, the differential entropy is increased with respect to the input signal (originally, ≈−51 and ≈−45), approaching values from ≈−10 up to 0, meaning that the H-proper structure of the QVAE works towards an increase in entropy and an improperness reduction in the input signal. Confirming this, the loss of entropy $P_{H}$ was drastically reduced with respect to the original signal, ranging from approximately 8 and 13 while the input entropy loss is around ≈51.

Hence, the H-proper QVAE can be approximated to learn improper distributions, on which it preserves the original structure of the signal while increasing the entropy of the latent space, at the cost of a limited improperness reduction. Although the slight modification on the distribution, the QVAE is still able to catch the correct statistics and to perform a good generation as well as a precise reconstruction, as Figure 2 and Figure 3 show.

To further assess the performance of the QVAE and provide a comparison with its real-valued counterpart, we test a real-valued VAE on the same signal benchmarks. We expect that the real-valued VAE performs similar to the QVAE in the proper case, but that the quaternion model outperforms the real-valued one when dealing with improper signals. Indeed, thanks to the quaternion algebra properties, the QVAE is able to learn intra-component correlations while the VAE do not catch them. Thus, in cases with no correlation among components i.e., proper signals, the two models should have similar performances. On the contrary, in the improper case where the correlation among components is higher the QVAE gains advantages from the quaternion algebra and obtains better performances. Results and details are reported in the Appendix A. However, thanks to the quaternion algebra properties, the QVAE has just 25% of free parameters with respect to its real-valued counterpart.

## 7. Conclusions

In this paper, we have conducted a thorough analysis on a H-proper quaternion-valued variational autoencoder (QVAE) under an information-theoretic perspective, investigating the entropy loss of the model due to the measure of improperness in the input signal. We have proved that the QVAE can be approximated for improper signals, for which it increases the entropy of the latent space by slightly reducing the improperness. Moreover, it is worth noting that, in each of our experiments, the properness is preserved also for nonlinear transformations brought by activation functions and not only for simple quaternion linear operations. As a conclusion, the QVAE is able to learn the correct distribution of both H-proper and improper random variables, while the measure of improperness, as well as the entropy of the latent space, can be calibrated through the KL divergence in the cost function.

## Figures and Tables

**Table 1:** Fundamental properties of H-proper random variables.

$E\left\{q_{\delta }^{2}\right\}=E\left\{q_{\epsilon }^{2}\right\}=\sigma^{2}$	$\;\;\;\forall \delta ,\epsilon =\left\{0,1,2,3\right\}$
$E\left\{q_{\delta }q_{\epsilon }\right\}=0$	$\;\;\;\forall \delta ,\epsilon =\left\{0,1,2,3\right\}$, δ≠ϵ
$E\left\{qq\right\}=-2\sigma^{2}$	$\;\;\;\forall \delta =\left\{0,1,2,3\right\}$
$E\left\{{\left|q\right|}^{2}\right\}=4\sigma^{2}$	$\;\;\;\forall \delta =\left\{0,1,2,3\right\}$

**Table 2:** In order to prove the measures and the assumptions presented in Section 5, we compute the average improperness coefficient *I* (16), the differential entropy H (20), the improperness measure P (19) and entropy loss $P_{H}$ (22) for different kinds of H-proper and improper signals. The entropy H is highest for the H-proper signals, while the improperness measure and coefficients are close to 0. As  is clear from P and $P_{H}$, there is a low entropy loss due to the improperness in the first two signals, while it is high for the last two, improper, random vectors.

Signal	*I*	H	P	$P_{H}$
P1. (proper)	0.0393	4.0177	0.0049	0.0049
P2. (proper)	0.0528	5.0174	0.0103	0.0103
I1. (improper)	1.0000	−51.6040	50.9298	51.0691
I2. (improper)	0.9999	−45.4454	51.3121	51.1843

**Table 3:** Average improperness coefficient *I* (16), differential entropy H (20), improperness measure P (19) and entropy loss $P_{H}$ (22) for different outputs from the QVAE (Recons stands for the reconstructed signal, Sample for the generated one) with different kinds of H-proper and improper signals. The QVAE preserves the structure of the signal, learning the properness and improperness nature of the input.

Signal	Out	*I*	H	P	$P_{H}$
P1. (proper)	Recons	0.0287	5.1519	0.0030	0.0029
	Sample	0.0537	2.3859	0.0096	0.0095
P2. (proper)	Recons	0.0640	3.4614	0.0124	0.0123
	Sample	0.1099	−0.2705	0.0348	0.0347
I1. (improper)	Recons	0.9989	0.3749	9.9700	9.9700
	Sample	0.9930	−3.6337	8.1208	8.1208
I2. (improper)	Recons	0.9998	−8.1100	13.0767	13.0767
	Sample	0.9964	−10.7147	12.1469	12.1469

**Figure A1:**
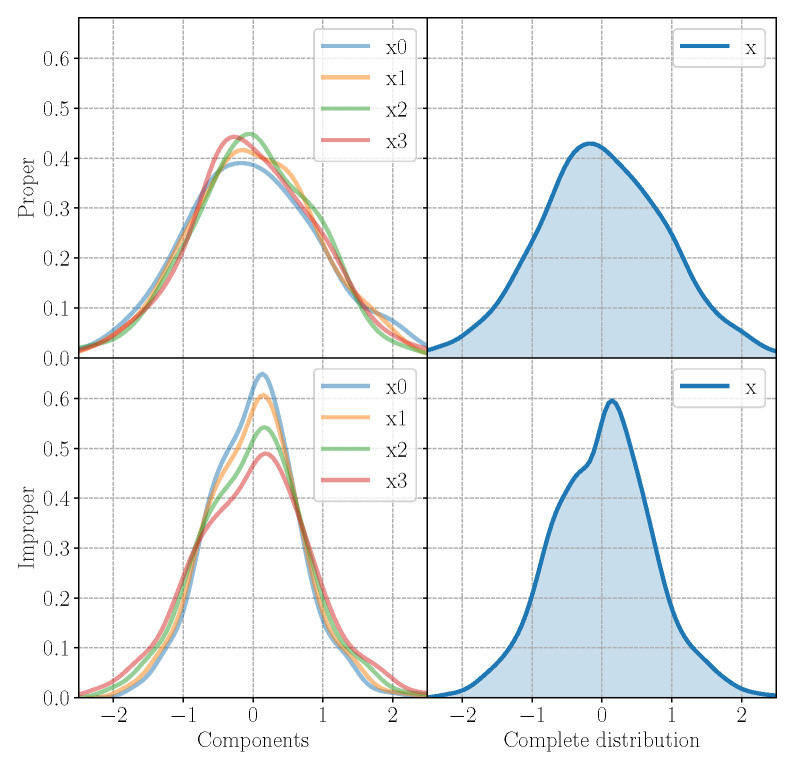
Generated samples from the real-valued VAE trained on the proper signal P1 and on the improper signal I2. These model generates good samples (upper figure), even if the variance is larger with respect to the QVAE.

**Figure A2:**
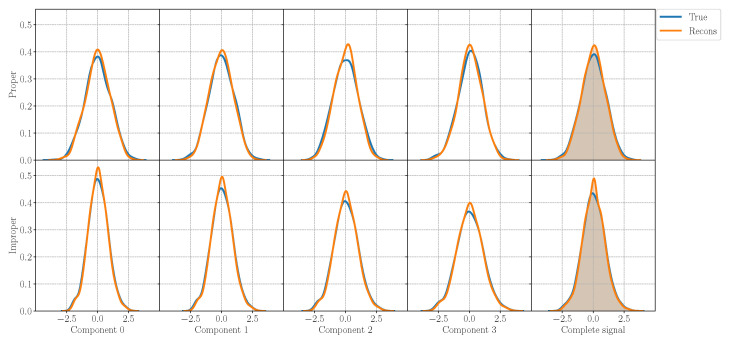
Reconstructed distributions from the real-valued VAE on signal P1 (proper) in the upper line and I2 (improper) in the lower line. Although the larger number of parameters, the VAE commits some errors in both the signal for the reconstruction task, while the QVAE is able instead to perfectly reconstruct original signals. The MSE values are 0.1237 for the proper signal and 0.0191 for the improper one.

## Data Availability

Not applicable.

## Appendix A. Evaluation of the Real-Valued VAE on Proper and Improper Signals

In order to further assess the performance of the H-proper QVAE, we perform a set of experiments with a real-valued VAE on the same benchmarks. In order to make a fair comparison, the hyperparameters are the same as for the QVAE, while the architecture is built with real-valued blocks. Moreover, the KL divergence is the standard divergence in [13]. However, due to the algebra in real domain, the VAE has 506,316 parameters while the QVAE has just 127,980, thus the proposed model in the quaternion domain has exactly the 25% of parameters with respect to its real-valued counterpart. Thus, the QvAE is able to learn the improperness of the input signal and to generate or to reconstruct precise signals while requiring 1/4 parameters and memory. Moreover, the QVAE is able to perfectly reconstruct the improper signal, as shown in Figure 3, while the real-valued VAE makes some errors. These results can be seen in Figure A1 and Figure A2. As we expect, the QVAE outperform the real-valued VAE when dealing with correlated signals i.e., improper cases, since the proposed method leverages the quaternion algebra properties, including the Hamilton product, to learn intra-components relations, while the real-valued VAE is not able to catch them. Thus, while for proper signals the performances between VAE and QVAE are similar, in the improper cases the QVAE has better performances thanks to the Hamilton product in its core blocks. Finally, the improperness measures and corresponding entropy loss for each of the signals considered are reported in Table A1.

**Table A1:** Average improperness coefficient *I* (16), differential entropy H (20), improperness measure P (19) and entropy loss $P_{H}$ (22) for different output from the real-valued VAE (Recons stands for the reconstructed signal, Sample for the generated one) with different kinds of H-proper and improper signals. As the QVAE, also the VAE, which is however 75% larger in terms of free parameters, is able to learn the improperness of the input.

Signal	Out	*I*	H	P	$P_{H}$
P1. (proper)	Recons	0.031	5.4547	0.0031	0.0030
	Sample	0.0697	5.4498	0.0148	0.0147
P2. (proper)	Recons	0.0396	4.8138	0.0050	0.0050
	Sample	0.9879	−21.2687	6.7205	6.7205
I1. (improper)	Recons	0.9983	−8.9158	14.0350	14.0350
	Sample	0.9995	−8.2952	16.1035	16.1035
I2. (improper)	Recons	0.9984	−9.0598	14.2591	14.2591
	Sample	0.9977	−9.2681	13.7240	13.7240
